# Optimization of isolation and transfection conditions of maize endosperm protoplasts

**DOI:** 10.1186/s13007-020-00636-y

**Published:** 2020-07-09

**Authors:** Yufeng Hu, Dalin Song, Lei Gao, Babatope Samuel Ajayo, Yongbin Wang, Huanhuan Huang, Junjie Zhang, Hanmei Liu, Yinghong Liu, Guowu Yu, Yongjian Liu, Yangping Li, Yubi Huang

**Affiliations:** 1 State Key Laboratory of Crop Gene Exploration and Utilization in Southwest China, Chengdu, China; 2 College of Agronomy, Sichuan Agricultural University, Chengdu, 611130 China; 3 College of Life Science, Sichuan Agricultural University, Ya’an, 625014 China

**Keywords:** Endosperm, Genes’ functions, Maize, Protoplast, Response surface, Transient system

## Abstract

**Background:**

Endosperm-trait related genes are associated with grain yield or quality in maize. There are vast numbers of these genes whose functions and regulations are still unknown. The biolistic system, which is often used for transient gene expression, is expensive and involves complex protocol. Besides, it cannot be used for simultaneous analysis of multiple genes. Moreover, the biolistic system has little physiological relevance when compared to cell-specific based system. Plant protoplasts are efficient cell-based systems which allow quick and simultaneous transient analysis of multiple genes. Typically, PEG-calcium mediated transfection of protoplast is simple and cost-effective. Notably, starch granules in cereal endosperm may diminish protoplast yield and integrity, if the isolation and transfection conditions are not accurately measured. Prior to this study, no PEG-calcium mediated endosperm protoplast system has been reported for cereal crop, perhaps, because endosperm cells accumulate starch grains.

**Results:**

Here, we showed the uniqueness of maize endosperm-protoplast system (EPS) in conducting endosperm cell-based experiments. By using response surface designs, we established optimized conditions for the isolation and PEG-calcium mediated transfection of maize endosperm protoplasts. The optimized conditions of 1% cellulase, 0.75% macerozyme and 0.4 M mannitol enzymolysis solution for 6 h showed that more than 80% protoplasts remained viable after re-suspension in 1 ml MMG. The EPS was used to express GFP protein, analyze the subcellular location of ZmBT1, characterize the interaction of O2 and PBF1 by bimolecular fluorescent complementation (BiFC), and simultaneously analyze the regulation of *ZmBt1* expression by ZmMYB14.

**Conclusions:**

The described optimized conditions proved efficient for reasonable yield of viable protoplasts from maize endosperm, and utility of the protoplast in rapid analysis of endosperm-trait related genes. The development of the optimized protoplast isolation and transfection conditions, allow the exploitation of the functional advantages of protoplast system over biolistic system in conducting endosperm-based studies (particularly, in transient analysis of genes and gene regulation networks, associated with the accumulation of endosperm storage products). Such analyses will be invaluable in characterizing endosperm-trait related genes whose functions have not been identified. Thus, the EPS will benefit the research of cereal grain yield and quality improvement.

## Background

The cereal endosperm, a storage tissue, serves as important source of nutrients for humans and animals, and industrial raw materials. In contrast to dicots such as *Arabidopsis thaliana*, which have a transitory endosperm, maize (*Zea mays* L.) and other cereals have persistent endosperm in their mature seeds [1, 2]. Cereal endosperm has received a lot of deliberate research attentions owing to its significance in agriculture. Like other angiosperms, maize endosperm is formed through the process of double fertilization, but undergoes the nuclear form of endosperm development (ESD) [2, 3], in which the primary endosperm nucleus divides repeatedly without cell wall formation [3, 4]. The endosperm cells, then, undergo mitosis and differentiated into four major specialized tissues: the transfer cells, aleurone layer, starchy endosperm, and embryo surrounding cells [4]. The transfer cells transport nutrient solutes from maternal tissues to the endosperm, while the starchy endosperm and aleurone layer are packed with starch granules, storage proteins and minerals. The embryo surrounding cells may be involved in communicating and transferring of nutrients between endosperm and embryo [4, 5]. In addition to the different number of specialized cell types the maize endosperm contains, it is relatively large, which makes it an excellent model for functional genomic studies [5].

Recently, advanced genomic and molecular tools have been applied to characterize a wide-array of specific genes’ functions associated with the ESD, such as the regulation of endosperm cell proliferation [6, 7] and accumulation of storage components [8–11]. Complex interactions exist among the key regulators and related genes involved in these processes. Although, there are existing advanced biotechnological systems for gene expression assays, most of them are expensive, time-consuming and involve complex protocols. These possibly explain why our understanding of molecular processes related with maize ESD is still far from complete, even though, vast information on endosperm-trait related genes and their regulatory factors are available. Thus, establishing a simple, inexpensive and highly effective endosperm cell-based system will deepen our understanding and prove valuable to the improvement of maize grain yield and quality.

Plant protoplasts are stable cell-based systems which have proven versatile for transient analysis of gene functions and regulations [12, 13]. Protoplasts are cells that have had their cell wall removed, and can easily take up and integrate exogenous nucleic acids [14]. Protoplast transfection is simple, stable, efficient and cost-effective as it can easily be manipulated by exogenous application of chemicals. These advantages of protoplast system, coupled with its high-resolution imaging, can be exploited to analyze and characterize gene functions and regulatory networks, particularly, in highly tissue-specific or cell-specific processes in plant such as promoter activation. The protoplast systems have been applied to investigate transient gene expression, protein subcellular localization, protein-DNA interaction, protein–protein interaction, cell signaling pathways in response to hormones, environmental cues, and transcriptional regulatory networks [15–21]. To date, protocols for protoplast isolation and transfection have been established in various plant models for different plant tissues such as leaves, roots, petals, cell suspension, seedlings, stems and sheaths [22]. The leaf protoplast systems show great utility, easier to isolate and manipulate, and are widely used in molecular studies. However, such systems may not be entirely applicable for all physiological processes and metabolic pathways, particularly for cell-specific biological processes [23].

Remarkably, there is no study that has reported endosperm-protoplast system (EPS) in cereal, partly because endosperm contains cells which accumulate starch grains. Previous studies have shown that starch granules are capable of diminishing protoplast yield and integrity [24–26]. However, if appropriate conditions for the isolation and transfection of protoplast for a specific plant tissue could be carefully measured, based on the proper understanding of the features of the plant tissue, such protoplast can be reliably utilized as experimental system for transient gene and transcriptome analyses [23]. In this study, we established appropriate conditions for efficient isolation and transfection of maize endosperm protoplast (MEP) to assay gene functions transiently, and validated the utilization of the maize EPS for protein immunoblotting, protein subcellular localization, protein–protein interaction by bimolecular fluorescent complementation (BiFC), and transient gene expression and regulatory analyses by qRT-PCR. We concluded that the EPS can be efficiently used to rapidly analyze large number of genes, which are associated with endosperm related traits.

## Results

### Protoplast yield response and optimization models of factors central to protoplast isolation in maize endosperm

**Fig. 1:**
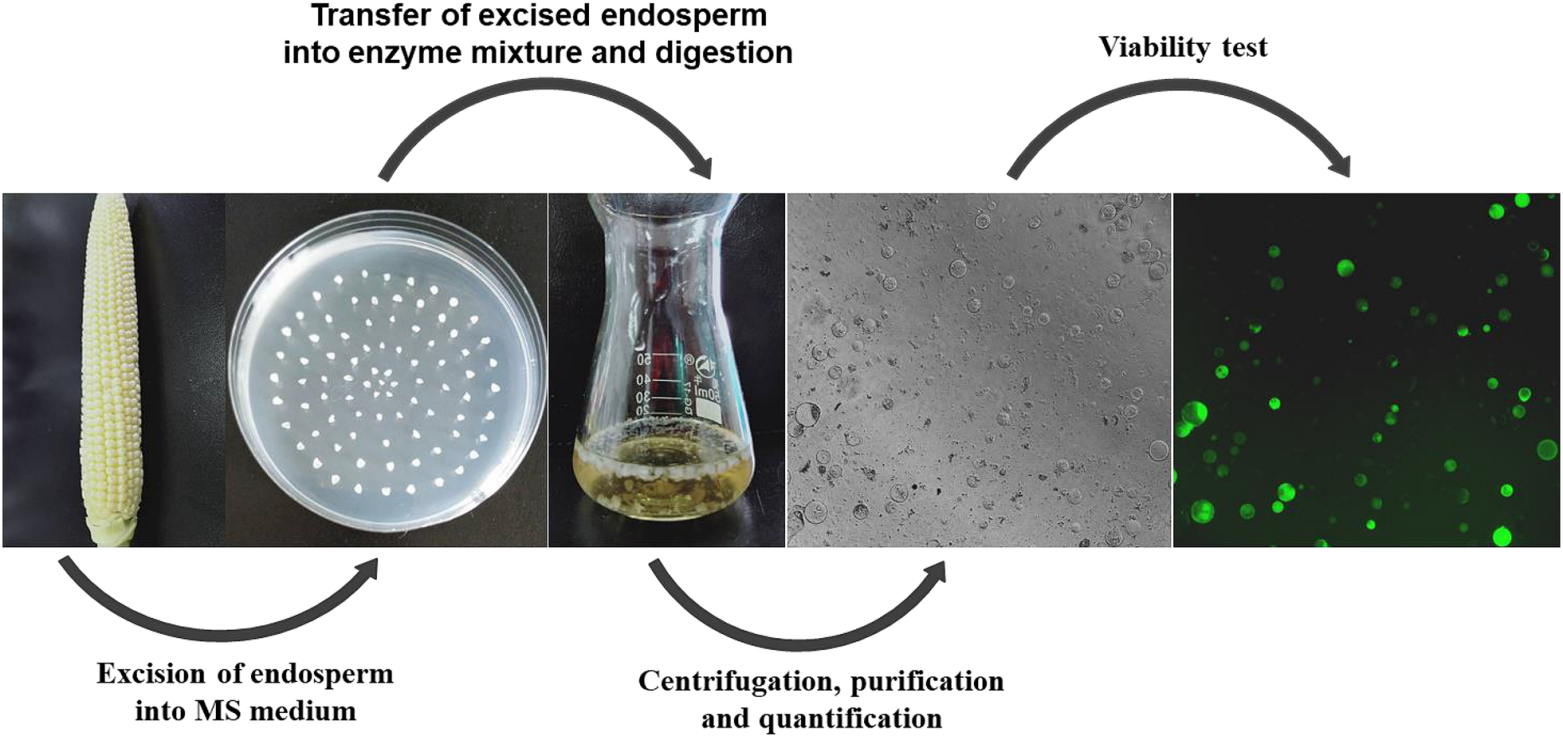
Schematic overview of protoplast isolation

The response surface method (RSM) is one of the experimental models for obtaining optimum settings for a range of factors affecting a response variable of interest. We investigated four factors with three coded levels by using Box-behnken design (BBD) to optimize protoplast yield response. The four experimental factors include cellulase concentration (*x*_*1*_), macerozyme concentration (*x*_*2*_), mannitol concentration (*x*_*3*_) and hydrolysis time (*x*_*4*_), each with three coded levels (−1, 0 and +1). The coded and corresponding actual levels of the experimental factors are given in Table 1. The major procedures involved in the isolation of the endosperm protoplast (EP) are illustrated in Fig. 1. The fluorescein diacetate (FDA) result showed that more than 80% of the protoplasts were viable after re-suspension in 1 ml MMG (Table 1). Protoplast yield (*y*) was used as the response variable for analyzing its relationship with the four investigated independent factors: *x*_*1*_, *x*_*2*_, *x*_*3*_ and *x*_*4*_. The quadratic polynomial response analysis of the experimental data described the relationship of protoplast yield with the four experimental factors (*x*_*1*_, *x*_*2*_, *x*_*3*_, and *x*_*4*_), according to Eq. (1). The ANOVA for the polynomial function is presented in Table 3.

1 $$\begin{aligned} Y &= \, - 9 9. 5 60 \, + { 39}. 4 80x_{1} + { 81}. 5 9 3x_{2} \\ &\quad+ { 62}. 30 4x_{3} + { 9}. 7 3 5x_{4} {-}{ 1}. 300x_{1} x_{2} \\&\quad + { 1}. 1 2 5x_{1} x_{3} {-} \, 0. 2 7 5x_{1} x_{4} + { 8}. 2 50x_{2} x_{3} \\ &\quad{-}{ 2}. 1 5 4* 10^{ 1 4} x_{2} x_{4} + { 1}. 6 5 6x_{3} x_{4} \\ &\quad{-}{ 12}. 3 9 3x_{1}^{ 2} {-}{ 56}. 1 7 3x_{2}^{ 2} - { 67}. 9 2 7x_{3}^{ 2}\\ &\quad {-} \, 0. 8 7 3x_{4}^{ 2} \\ \end{aligned}$$

**Table 1:** Experimental and coded levels used in box-behnken design for studying the effects of cellulase (x_1_), macerozyme (x_2_), mannitol (x_3_) and hydrolysis time (x_4_) on yield of isolated protoplasts along with the predicted mean and observed responses, and FDA results

Experimental factor	Coded symbol	Coded variable levels									
		Low	Centre	High							
		−1	0	+1							
Cellulase (%)	X_1_	1.00	1.50	2.00							
Macerozyme (%)	X_2_	0.50	0.75	1.00							
Mannitol	X_3_	0.40	0.60	0.80							
Hydrolysis time (h)	X_4_	4	6	8							

Test Number	Coded levels				Actual levels				Response		
									Yield, Y (×10^6^ cells/ml)		FDA
	X_1_	X_2_	X_3_	X_4_	*X*_*1*_	*X*_*2*_	*X*_*3*_	*X*_*4*_	*Observed Y*	*Predicted Y*	(%)
*Coded levels for the four factors selected for protoplast isolation*											
1	+1	+1	0	0	2.00	1.00	0.60	6	0.95	1.52	92.3
2	+1	−1	0	0	2.00	0.50	0.60	6	1.45	1.68	93.9
3	−1	+1	0	0	1.00	1.00	0.60	6	1.25	1.49	90.9
4	−1	−1	0	0	1.00	0.50	0.60	6	1.10	1.00	95.8
5	+1	0	+1	0	2.00	0.75	0.80	6	1.55	2.22	95.1
6	+1	0	−1	0	2.00	0.75	0.40	6	2.70	2.56	97.2
7	−1	0	+1	0	1.00	0.75	0.80	6	1.70	1.65	94.6
8	−1	0	−1	0	1.00	0.75	0.40	6	3.30	2.43	96.9
9	+1	0	0	+1	2.00	0.75	0.60	8	1.70	1.02	90.3
10	+1	0	0	−1	2.00	0.75	0.60	4	2.85	2.21	95.1
11	−1	0	0	+1	1.00	0.75	0.60	8	0.85	1.22	90.5
12	−1	0	0	−1	1.00	0.75	0.60	4	0.90	1.31	92.0
13	0	+1	+1	0	1.50	1.00	0.80	6	3.00	2.02	96.0
14	0	+	−1	0	1.50	1.00	0.40	6	1.70	1.75	91.8
15	0	−1	+1	0	1.50	0.50	0.80	6	1.35	1.03	94.1
16	0	−1	−1	0	1.50	0.50	0.40	6	1.70	2.41	91.5
17	0	+1	0	+1	1.50	1.00	0.60	8	0.95	0.79	96.4
18	0	+1	0	−1	1.50	1.00	0.60	4	1.15	1.43	92.3
19	0	−1	0	+1	1.50	0.50	0.60	8	1.10	0.62	91.7
20	0	−1	0	−1	1.50	0.50	0.60	4	1.30	1.26	93.8
21	0	0	+1	+1	1.50	0.75	0.80	8	1.30	1.88	95.8
22	0	0	+1	−1	1.50	0.75	0.80	4	1.10	1.20	97.3
23	0	0	−1	+1	1.50	0.75	0.40	8	0.75	1.12	93.3
24	0	0	−1	−1	1.50	0.75	0.40	4	3.20	3.08	93.8
25	0	0	0	0	1.50	0.75	0.60	6	7.75	8.03	93.3
26	0	0	0	0	1.50	0.75	0.60	6	8.50	8.03	93.9
27	0	0	0	0	1.50	0.75	0.60	6	7.60	8.03	91.8
28	0	0	0	0	1.50	0.75	0.60	6	8.25	8.03	96.7
29	0	0	0	0	1.50	0.75	0.60	6	8.05	8.03	94.1

The quadratic model for the ANOVA was highly significant (p < 0.0001), suggesting that the model for the regression terms was adequate, and that a higher order model would not be needed. The R-square value (0.9687) further established the reliability of the model, which explained 96.87% of variation in the experimental data. Intrestingly, the lack of fit relative to pure error was not significant, indicating that the experimental data fitted well to the design model.

The magnitudes of the regression coefficients of the linear and quadratic main effects were larger relative to the interaction effect of the experimental variables (Eq. 1), suggesting that the linear and quadratic main effects were more important than for the interaction effect of the factors. The ANOVA results confirmed this implication, as the mean squares for the linear and quadratic main effects were highly significant (p < 0.0001) for all the factors, except linear main effect for the hydrolysis time which was not significant (Table 3). Furthermore, the regression coefficients of the linear main effect of the four factors: cellulase and macerozyme (hydrolytic enzymes), mannitol and duration of hydrolysis, indicated positive influence on the yield of the isolated protoplasts (Eq. 1). The hydrolytic enzyme, macerozyme (x2), had the strongest direct impact, followed by the mannitol (osmotic solute) with the least influence by hydrolysis duration. Moreover, the protoplast yield exhibited negative quadratic response to the increased levels of the cellulase, macerozyme, mannitol and duration of hydrolysis, as indicated by the negative values of the quadratic coefficients in the polynomial function (Eq. 1). Therefore, the optimal region for each independent variable is a maximum rather than minimum (i.e. the curvature is convex). The significance of the curvature (quadratic term) for each factor, indicates that the experimental region may be close to the optimum. This suggests the need to simultaneously determine the optimal settings for hydrolysis time and concentrations of cellulase, macerozyme and mannitol that will result in protoplast yield optimization. In contrast to the linear main effect, mannitol indicated the largest negative quadratic effect on the protoplast yield, followed by the hydrolytic enzymes. This suggests that a slight or unit increase in the concentration of either mannitol or hydrolytic enzyme(s) above the optimal level, will result in a considerable reduction in protoplast yield. All the interaction effects were not significant, indicating that 3D surface plot of the experimental factors would not be necessary.

**Fig. 2:**
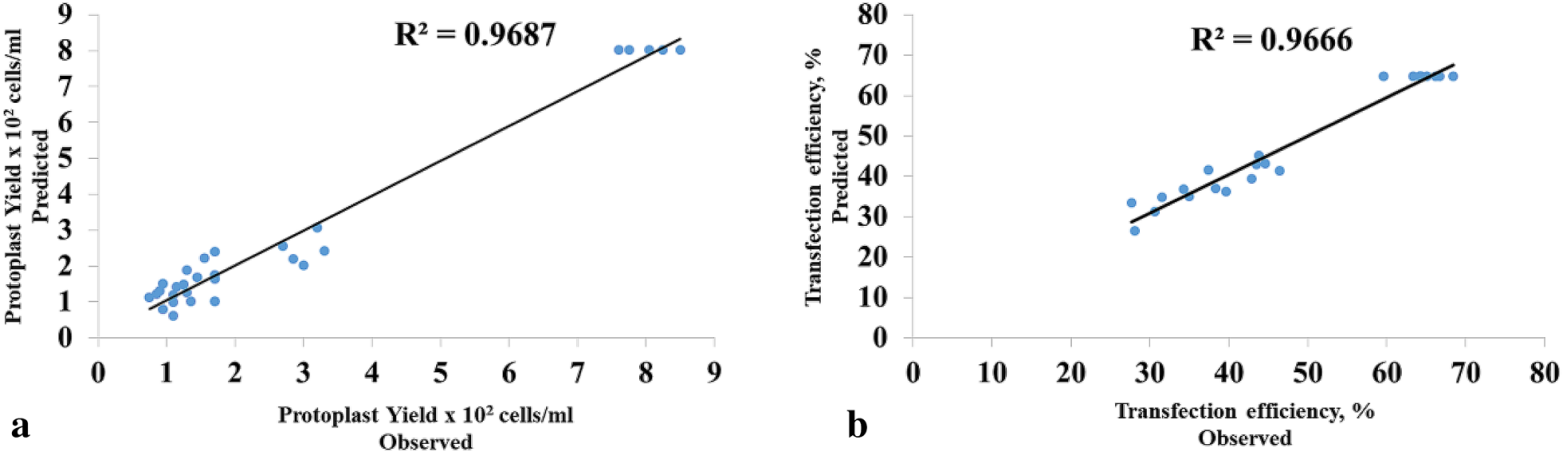
Normal plots for predicted Vs observed protoplast yields and transfection efficiencies. **a** Normal plot for predicted Vs observed protoplast yields. **b** Normal plot for predicted Vs observed protoplast transfection efficiencies

The experimental levels ranged from 1 to 2% for cellulase, 0.5 to 1% for macerozyme, 0.4 to 0.8 M for mannitol and 4 to 8 h for hydrolysis time (Table 1). The observed protoplast yield responses varied from 0.75 × 10^6^ to 8.5 × 10^6^ cells/ml, while predicted protoplast yield responses were in the range of 0.62 × 10^6^ to 8.03 × 10^6^ cells/ml. The predicted responses reasonably matched and were consistent with the experimental results of protoplast yields (Fig. 2a, Table 1). The optimisation analysis revealed 1.0% (w/v) cellulase, 0.75% (w/v) macerozyme, 0.40 M mannitol and 6 h hydrolysis time as the optimal levels, that would result in optimal protoplast yield response of 2.43 × 10^6^ protoplast cells/ml (Table 3).

### Transfection efficiency response of MEP and optimization of PEG-Ca^2+^ mediated transfection conditions

In order to establish a suitable combination of factors, for the optimum response of protoplast efficiency in maize endosperm, central composite design (CCD) was successfully used to estimate the number of tests required for the PEG-calcium mediated transfection experiment. The experiment involved three factors including protoplast concentration (x_1_), total plasmid DNA (x_2_) and endosperm age (x_3_). In all a total of twenty-three tests (these included 9 center points) were performed. The transfection efficiency (*Y*_*1*_) data obtained were subjected to response surface quadratic model. The results revealed a regression model according to Eq. (2). The response surface quadratic model for the ANOVA was highly significant (p < 0.0001) with R-square value of 0.9666 (Table 4). The mean square for lack of fit of the model, however, was not significant. These, altogether, indicate that the model is reliable and adequate to describe our data, and that higher order model is not required.

2 $$\begin{aligned} Y_{1}^{2} &= 4196.5678 + 100.3229x_{1} + 90.3972x_{2} \\ &\quad- 180.9542x_{3} - {\mkern 1mu} 0.6x_{1} x_{2} + 26.7x_{1} x_{3} \\ &\quad - 161.5x_{2} x_{3} - 824.1365x_{1}^{2} \\ &\quad- 884.1457x_{2}^{2} - 1127.7132x_{3}^{2} \\ \end{aligned}$$

The ANOVA results showed highly significant (p < 0.0001) linear and quadratic main effects for all the factors, except, linear main effect for x_2_ and x_3_ that were not significant. Moreover, all the interactions involving the three factors were not significant (Table 4). Consistent with the ANOVA results, both the linear and quadratic regression terms had larger coefficients relative to the coefficients of the interaction terms. This indicates the superior influence of both the linear and quadratic main effects on the MEP transfection efficiency response. Strikingly, the MEP transfection efficiency showed positive linear response to protoplast concentration and total plasmid DNA, but negative linear response to endosperm age. Specifically, this result shows that the efficiency of transfection significantly drops with increased endosperm age. This probably is associated with increased starch granule accumulation as endosperm develops [24–26].

Although, transfection efficiency response was largely determined by the linear and quadratic main effects, but the quadratic main effect showed higher significant influence, as indicated by the magnitude of the coefficients of the quadratic terms. Notably, the MEP transfection efficiency displayed negative quadratic surface response to protoplast concentration (x_1_), total plasmid DNA (x_2_) and endosperm age (x_3_) (Eq. 2). Thus, determining the ideal settings of the three factors is critical to obtaining an optimal transfection efficiency response. Since the interaction mean squares for all the factors were not significant, point optimization analysis rather than 3D surface plot, will prove appropriate to determine the optimal levels of the experimental factors. The various levels of protoplast concentration, total plasmid DNA and endosperm age varied from 0.5 to 2.5 × 10^6^ protoplast cells/ml, 5 to 15 µg, and 6 to 10 DAP, respectively (Table 2). Meanwhile, the protoplast transfection efficiencies were in the range of 27.7 to 68.4% for observed and 26.6 to 64.8% for predicted responses. There is consistency in the observed and predicted efficiencies of the transfected protoplasts (Fig. 2b, Table 2). We performed point optimization analysis to determine the optimum transfection efficiency response. The optimum transfection efficiency was ~ 65% with 95% confidence interval of 63– 68% (Table 4). The optimal levels of protoplast concentration, total plasmid DNA and endosperm age that would result in optimum transfection efficiency of 65% were 1.5 × 10^6^ protoplast cells/ml, 10 µg plasmid DNA and 8 DAP endosperm (Table 4).

**Table 2:** Experimental and coded levels used in central composite design for studying the effects of protoplast concentration (x_1_), total plasmid DNA (X_2_), and endosperm age (x_3_) on protoplast transfection efficiency along with the predicted mean and observed responses

Experimental factor	Coded symbol	Coded variable levels						
		Lowest	Low	Centre	High	Highest		
		-α (−1.68)	−1	0	+1	+α (+1.68)		
Protoplast Conc. (× 10^6^) cells/ml	X_1_	0.5	1.0	1.5	2.0	2.5		
Total plasmid DNA (µg)	X_2_	5	7	10	13	15		
Endosperm age (DAP)	X_3_	6	7	8	9	10		

Test number	Coded levels			*Actual levels*			Response	
							Transfection efficiency, Y_1_ (%)	
	X_1_	X_2_	X_3_	*X*_*1*_	*X*_*2*_	*X*_*3*_	Observed Y_1_	Predicted Y_1_
*Coded levels for the three factors selected for protoplast transfection*								
1	+1	+1	+1	2	13	9	35.0	35.1
2	+1	+1	−1	2	13	7	44.6	43.2
3	+1	−1	+1	2	7	9	38.3	37.1
4	+1	−1	−1	2	7	7	34.3	36.9
5	−1	+1	+1	1	13	9	30.6	31.3
6	−1	+1	−1	1	13	7	37.4	41.5
7	−1	−1	+1	1	7	9	27.7	33.5
8	−1	−1	−1	1	7	7	31.5	34.9
9	+1.68	0	0	2.5	10	8	43.8	45.2
10	−1.68	0	0	0.5	10	8	46.4	41.3
11	0	+1.68	0	1.5	15	8	43.5	43.0
12	0	−1.68	0	1.5	5	8	42.9	39.4
13	0	0	+1.68	1.5	10	10	28.1	26.6
14	0	0	−1.68	1.5	10	6	39.6	36.3
15	0	0	0	1.5	10	8	64.3	64.8
16	0	0	0	1.5	10	8	63.3	64.8
17	0	0	0	1.5	10	8	68.4	64.8
18	0	0	0	1.5	10	8	66.2	64.8
19	0	0	0	1.5	10	8	64.4	64.8
20	0	0	0	1.5	10	8	65.1	64.8
21	0	0	0	1.5	10	8	64.2	64.8
22	0	0	0	1.5	10	8	59.6	64.8
23	0	0	0	1.5	10	8	66.7	64.8

### Application of MEP system in protein immunoblotting, protein subcellular localization and bimolecular fluorescence complementation (BiFC) assays

**Fig. 3:**
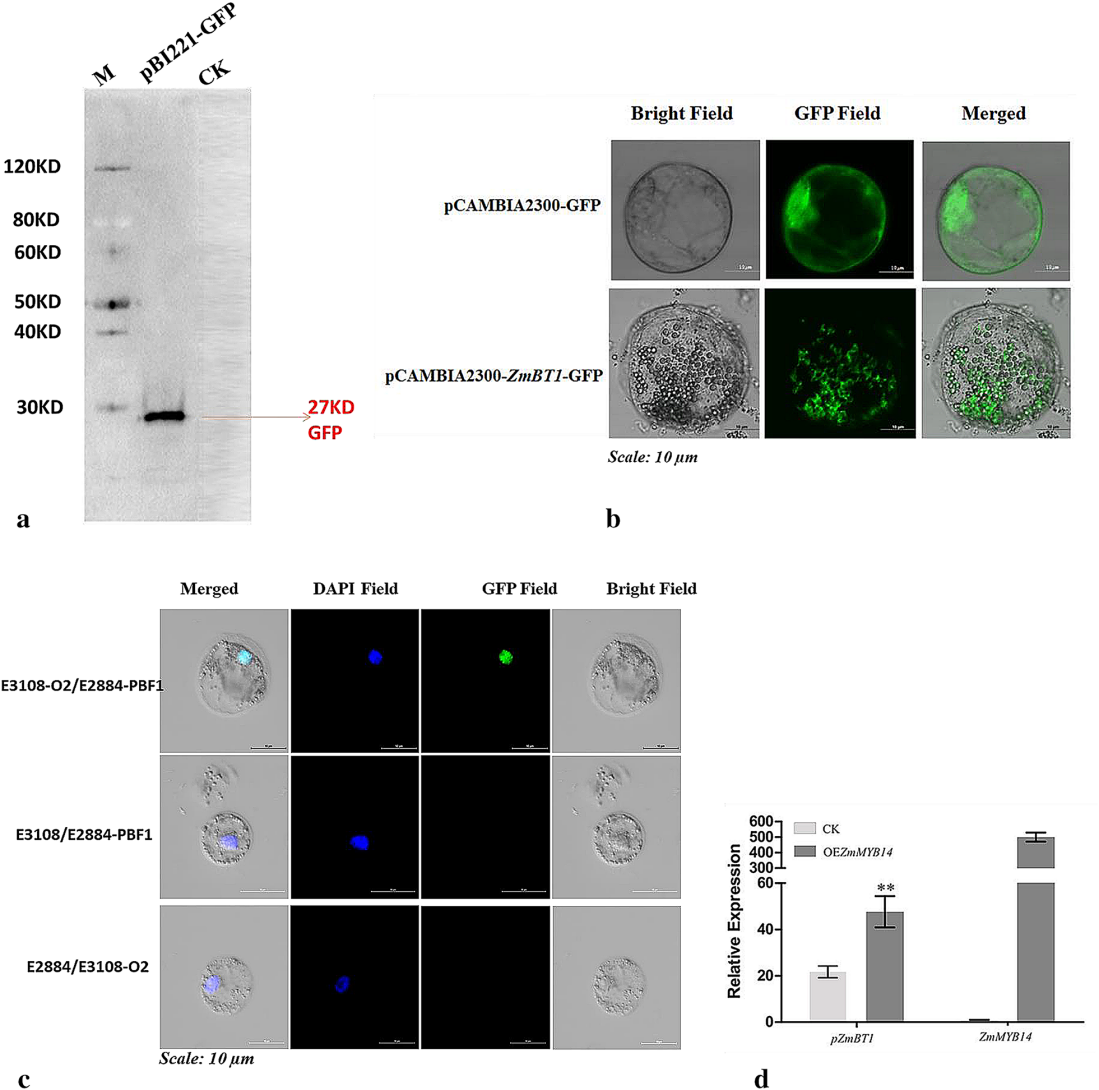
Application of endosperm protoplast system in protein immunoblotting, subcellular localization, BiFC and promoter activation analysis. **a** Expression of GFP protein, **b** subcellular localization of ZmBt1, **c** interaction of O2 and PBF1 proteins by BiFC assay, **d** positive activation of ZmBt1 promoter by ZmMYB14; mean ± SE, *t* test, p < 0.001 (**), n = 3

In order to be sure, if our EPS can be used as a model system to study protein immunoblotting, the pBI221-*GFP* construct was transfected into the EPS. Un-transfected protoplast was used as control (CK). The CK had no band, whereas, the transfection system containing the pBI221-*GFP* construct showed band of about 27KD, approximately the size of GFP protein (Fig. 3a). These results suggest that the EPS is suitable and can be used for protein expression.

Also, we investigated the subcellular localization of maize Brittle 1 (ZmBT1) protein using maize endosperm protoplast, to explore the empirical use of the EPS in molecular study of protein localization. The ZmBT1 protein transports adenosine diphosphate glucose (ADPG) from the cytosol to the amyloplasts of the maize endosperm for starch synthesis, and has been shown to be localized in the amyloplast envelope membrane [27, 28]. The protein expression vector, 2300-*ZmBt1*-*GFP*, was transfected into the EP by PEG-calcium mediated transfection method. The empty vector, 2300-*GFP*, was also transfected into the protoplasts as control system. Consistent with the previous studies, the fusion protein vector, 2300-*ZmBt1*-*GFP*, was correctly expressed and uniformly localized at the plasma membrane of the amyloplasts (Fig. 3b). Conversely, the control system showed fluorescent signal in the entire cell (Fig. 3b). These results indicate that the EPS can be applied in protein localization studies.

Furthermore, the EPS was employed to investigate protein–protein interaction by BiFC assay. We investigated the interaction of the two transcription factors (TFs), O2 and PBF1, in EPS using the BiFC assay. The two proteins are endosperm-specific TFs that cooperatively network the transcriptional regulation of genes involved in starch and storage (zein) protein accumulation [29]. The expression vectors, E2884-*PBF1* and E3108-*O2*, were constructed and co-transfected into the EPS. The vector pairs; E3108/E2884-*PBF1* and E2884/E3108-*O2* were also transfected into protoplasts for control experiments. The transfected protoplasts were stained with 4, 6-diamidino-2-phenylindole (DAPI) for fluorescence microscopy. The observed blue fluorescence for all the transfected systems revealed that the TFs were localized in the nucleus (Fig. 3c). No green fluorescent (GFP) signal was detected for the controls. However, the transfection system containing the constructs of the two proteins (O2 and PBF1) showed sharp green fluorescent signal, indicating the nuclear interaction of the two proteins (Fig. 3c).

### Transient expression of *ZmMYB14* in EPS and its influence on the expression of *ZmBt1*

The protoplast transient expression assays are invaluable tools for conducting cell-based experiments, using high-throughput approaches to the analyses of gene functions and regulatory networks. Relative to biolistic transient assays, the protoplast transient systems are more effective and provide greater functional analysis of genes [30, 31]. Starch, the main storage component of maize endosperm, is synthesized in the amyloplasts. The ZmBT1 has been reported to be functionally involved in the transport of Adenosine diphosphate glucose, from the cytosol to the amyloplasts for starch biosynthesis. Mutation of ZmBT1 can significantly limit the rate of starch biosynthesis in maize endosperm [27]. Recently, we found a novel endosperm-specific TF, ZmMYB14, which enhanced the promoter activity of *ZmBt1* [32]. To test whether the EPS can be effectively used for regulatory network analysis, we transfected pUbi-*ZmMYB14* vector, which was previously used in our study [32], into the endosperm protoplasts to analyze the transient expression of ZmMYB14, and its regulatory influence on the expression of *ZmBt1*. We extracted total RNA directly from the transfected protoplasts and performed qRT-PCR. The *TXN* gene was used as internal control, and un-transfected protoplast as the CK experiment. As shown in Fig. 3d, the ZmMYB14 was successfully over-expressed in the transfected EPs. Consistent with our previous study [32], the expression of ZmBT1 in the transfected protoplast cells was significantly increased by the over-expression of ZmMYB14 relative to the CK (Fig. 3d). These results indicate that the EPS can be suitably used, for the transient expression of foreign genes and analysis of gene regulatory networks.

## Discussion

Protoplast transient expression systems are versatile and effective molecular tools for studying gene functions and various cell-specific processes in plants. Several protocols have been established for isolating protoplasts from different plant tissues, as well as manipulating the isolated protoplast for studying various plant biological functions in vitro [22]. However, no study has reported endosperm protoplast-based system in cereal. This may be possibly because endosperm accumulates high starch content, which can significantly reduce protoplast yield and integrity [24–26]. Nevertheless, our study described effective and feasible protocols and determined optimal conditions, for MEP isolation and polyethylene glycol-calcium mediated transfection.

For the purpose of reliability and reproducibility, reasonable yield of healthy protoplast is important [22]. In our study, more than 80% protoplasts remained viable after re-suspension in 1 ml MMG. Our results showed that the models employed to the studying of protoplast isolation and transfection in maize endosperm were appropriate and satisfactory. This suggests that our data were accurately and sufficiently described. Our results revealed that cellulase and macerozyme (hydrolytic enzymes), mannitol and hydrolysis time had strong influence on protoplast yield. Several studies have reported that various factors including enzyme mixture (e.g. cellulase and macerozyme), osmotic solutes such as mannitol, and time of exposure of tissue to enzyme solution are crucial for isolation of protoplast from plant tissues [13, 22, 23, 31]. Moreover, protoplast concentration, total plasmid DNA and endosperm age, all had striking influence on protoplast transfection efficiency. This is consistent with previous studies that efficiency of transfected protoplast cells is largely dependent on type and age of plant tissues, and ratio of viable cells to exogenous DNA [23, 31]. Remarkably, the quadratic terms for all the parameters investigated for both protoplast isolation and transfection studies were highly important. This strongly indicated the need to investigate suitable conditions for optimal protoplast yield and transfection efficiency in maize endosperm. A proper combination of enzyme mixture, solute concentration for maintenance of cell turgor pressure and hydrolysis time is key to adequate protoplast yield [22]. The results of this study determined that, for protoplast isolation, 1% (w/v) cellulase, 0.75% (w/v) macerozyme, 0.40 M mannitol, and 6 h hydrolysis duration (Table 3) were appropriate for optimal protoplast yield from maize endosperm. The optimal conditions reported here for protoplast isolation from maize endosperm were slightly different from those described for other tissues such as leaf in maize and other model crops by other researchers [13, 22, 23, 31]. In our study, the observed protoplast yields from maize endosperm varied between 7.5 × 10^5^ and 8.5 × 10^6^. This range, however, is comparable with protoplast yield reported for other plant tissues in previous studies [13, 23, 31, 33]. While there are several methods of protoplast transfection, PEG mediated transfection technique is simple, efficient, and compatible with many protoplast system, as well improves transfection efficiency [34]. For a reliable transfection efficiency, conditions such as protoplast culture density and DNA to protoplast ratio must be optimized [30]. In this study, optimization analysis of transfection efficiency revealed that 1.5 × 10^6^ protoplast cells/ml, 10 µg plasmid DNA and endosperm age of 8 DAP were suitable parameters for high protoplast transfection efficiency in maize endosperm (Table 4). It is worthwhile to note that, endosperm age indicated negative linear and quadratic effects on the MEP transfection efficiency. This implies that only one optimal point is possible; that is endosperm age either below or above this optimal point, will result in considerable reduction in transfection efficiency. Our optimization analysis results revealed 8 DAP as an optimal explant age, for efficient protoplast transformation system in maize endosperm. Thus, the use of endosperms below or above 8 DAP is not desirable for efficient protoplast isolation or transfection. Two possible reasons for this observation are: (i) limited subcellular proliferation that may be associated with endosperms below 8 DAP, and (ii) higher accumulation of storage metabolites (such as starch) in endosperms above 8 DAP. In maize, upon fertilization, the primary endosperm cell undergoes rapid nuclear proliferation without cell wall formation, which takes place at about 1–3 DAP. Subsequently, the proliferated nuclei become cellularize with formation of cell wall materials at around 3–6 DAP. Beginning from around 6 DAP, the maize endosperm become differentiated into different cell types that become recognisable cytologically by 8 DAP. Commencing from 8 DAP, the central part of the endosperm gradually become filled with storage starch and proteins. Accumulation of starch, however, peaks steadily after this period [4, 35, 36]. Studies have shown that higher accumulation of starch [24–26] and limited subcellular proliferation in young plant tissues [37], can considerably diminish protoplast yield, viability and transfection. Thus, excised endosperm at 8 DAP, as established in our study, is optimally suitable for the isolation and transfection of MEP.

**Table 3:** Quadratic model ANOVA and optimization for protoplast yield

Source	Sum of squares	DF	Mean square	F-value	p-value	R-square
Model	178.89	14	12.78	30.91	*< 0.0001 S*	*0.9687*
X_1_-Cellulase Conc.	35.31	1	35.31	85.4	< 0.0001	
X_2_-Pectinase Conc.	41.06	1	41.06	99.32	< 0.0001	
X_3_-Mannitol	20.6	1	20.6	49.83	< 0.0001	
X_4_-Hydrolysis time	0.32	1	0.32	0.78	0.3924	
X_1_X_2_	0.11	1	0.11	0.26	0.6211	
X_1_X_3_	0.05	1	0.05	0.12	0.7316	
X_1_X_4_	0.3	1	0.3	0.73	0.4067	
X_2_X_3_	0.68	1	0.68	1.65	0.2203	
X_2_X_4_	2.84E−14	1	2.84E−14	6.87E−14	1	
X_3_X_4_	1.76	1	1.76	4.25	0.0584	
X_1_^2^	62.27	1	62.27	150.61	< 0.0001	
X_2_^2^	79.95	1	79.95	193.38	< 0.0001	
X_3_^2^	47.89	1	47.89	115.82	< 0.0001	
X_4_^2^	79.1	1	79.1	191.32	< 0.0001	
Residual	5.79	14	0.41			
Lack of Fit	5.26	10	0.53	3.94	*0.0990 NS*	
Pure error	0.53	4	0.13			
Corrected total	184.68	28				

Factor	Name	Optimal level	Experimental level range			
		Optimum	Low	Centre	High	
Optimization for yield of isolated maize endosperm protoplast						
X_1_	Cellulase Conc.	*1*	1	1.5	2	
X_2_	Pectinase Conc.	*0.75*	0.5	0.75	1	
X_3_	Mannitol	*0.4*	0.4	0.6	0.8	
X_4_	Hydrolysis time	*6*	4	6	8	

Predicted response	Protoplast yield	Standard deviation	SE mean	95% CI low	95% CI high	
	Optimal response					
	× 10^6^					
	2.43	0.643	0.491	1.38	3.49	

*N* Significant, *NS* non-significant, *SE* standard error, *CI* confidence interval

**Table 4:** Quadratic model ANOVA and optimization for protoplast transfection efficiency

Source	Sum of squares	DF	Mean square	F-value	p-value	R-Square
Model	43,683,750	9	4,853,750	41.82,179	< *0.0001 S*	*0.9666*
X_1_-Protoplast Conc.	8,769,727	1	8,769,727	75.56,336	< 0.0001	
X_2_-Total Plasmid DNA	22,592.06	1	22,592.06	0.194662	0.666,314	
X_3_-Endosperm Age	55,999.63	1	55,999.63	0.482514	0.499,513	
X_1_X_2_	2.88	1	2.88	2.48E−05	0.996101	
X_1_X_3_	5703.12	1	5703.12	0.04914	0.82801	
X_2_X_3_	208,658	1	208658	1.797878	0.202929	
X_1_^2^	10,755,781	1	10,755,781	92.67597	< 0.0001	
X_2_^2^	12,379,165	1	12,379,165	106.6637	< 0.0001	
X_3_^2^	20,139,145	1	20,139,145	173.5267	< 0.0001	
Residual	1,508,753	13	116,057.9			
Lack of Fit	713,758.2	5	142,751.6	1.436,504	*0.0385 NS*	
Pure Error	794,994.8	8	99,374.34			
Corrected Total	45,192,503	22				

Factor	Name	Optimal level	Experimental level range			
		Optimum	Lowest	Highest		
Optimization for protoplast transfection efficiency						
X_1_	Protoplast concentration (10^6^/ml)	1.5	0.5	2.5		
X_2_	Total plasmid DNA (µg)	10	5	15		
X_3_	Endosperm Age (DAP)	8	6	10		

Predicted response	Transfection efficiency (%)	Standard deviation	95% CI low	95% CI high		
	64.8	0.26	63	68.4		

*N* Significant, *NS* non-significant, *SE* standard error, *CI* confidence interval

The result of our PEG-calcium mediated transfection system showed transfection efficiencies which ranged from 63 to 68% (Table 2), with optimal efficiency of 65% (Table 4). A decade ago, *Agrobacterium tumefaciens*-mediated transfection of in vitro cultured endosperm in maize was reported. Following this method, the proportion of transfected cells of the aleurone layer varied between 10 and 20% [38]. Compared to the *Agrobacterium tumefaciens*-mediated transfection method, our PEG-calcium mediated transfection technique is more efficient and reliable, as the transfection efficiency is higher (63–68%). For protoplast system, a transfection efficiency higher than 50% was recommended and considered reliable to obtain reproducible data for transient expression system [31]. Thus, our transfection method is better and reproducible, and the established transfection conditions are reliable as indicated by the optimal transfection efficiency level of 65%. Thus, we recommend our established conditions for the isolation and PEG-calcium mediated transfection method for MEP system.

Freshly isolated protoplasts reserve their cell uniqueness, show great transfection efficiency and have been proven to be a physiological and versatile cell system, for studying gene functions and analysis of gene regulatory networks in plant [30]. Endosperm protoplast has not been currently employed as experimental system for transient gene and transcriptome analysis. Our study, however, showed that protoplast-based system can be used to study the functions of genes and proteins associated with endosperm related traits. For protein expression, we investigated the expression of GFP protein in EPS. The expressed GFP protein showed that the EPS is suitable for protein immunoblotting. In addition, the consistency of plastidial membrane localization of ZmBT1 protein with previous studies, indicated the applicability of the EPS to protein subcellular localization analysis.

Also, the usefulness of EPS to BiFC for protein–protein interaction assay was verified. Interaction of two TFs, O2 and PBF1, was investigated in this study. Both proteins had been reported to modulate starch and protein accumulation during grain filling stage of maize kernel development, through transcriptional regulation of their target genes [29]. Consistent with previous study, we confirmed the nuclear localization and interaction of the two TFs. Our observation of interaction of the two proteins, shows the suitability and effectiveness of our EPS as a versatile system for transient gene analysis. Transient gene analysis by using a high level protoplast transfection efficiency (greater than 50%) and improved GFP marker, often results in a greater physiological relevance to plants, when compared to data obtained from biolistic or heterologous cell systems [30, 31]. The high transfection efficiency (65%) obtained in this study, with the use of *GFP* fluorescent marker, underscores the suitability of the EPS for studying protein–protein interaction by BiFC assays. Finally, the EPS was used for transient expression of ZmMYB14 and transcriptional regulatory analysis of *ZmBt1*. These results were consistent with the previous study [32]. It is worthy to note that Both ZmMYB14 and ZmBT1 were expressed in one protoplast transfection system, indicating that multiple genes can be analyzed simultaneously in the EPS. This shows the advantage of the EPS for transient gene expression over biolistic system, which can only be used to test one gene at a time. In addition, biolistic system is expensive, involves complex technical know-how and requires a lot of experience. Whereas, the EP transient system is simple, stable, efficient and cost-effective. These advantages of EP transient system, coupled with its higher transfection efficiency, can be effectively exploited to simultaneously analyze large number of endosperm-trait related genes. More recently, the high throughput advantages of protoplast system were exploited and demonstrated by Gao et al. [39], where maize mesophyll protoplast was applied in analyzing protein localization, protein–protein interactions and transient expression of genes and regulators associated with benzoxazinoid biosynthesis. Therefore, the EPS developed in our study provides another functional genomic tool in maize for transient analysis of genes and protein functions, particularly, for endosperm-specific genes and regulatory networks.

## Conclusions

In summary, we developed effective protocols and optimized conditions for protoplast isolation and transfection systems in maize endosperm. We showed that the EP can be used as a model system to study protein immunoblotting, protein subcellular localization, bimolecular fluorescent complementation assays for protein–protein interaction, and transient gene expression assays and analysis of gene regulatory networks. The MEP system proved to be an effective tool for rapid analysis vast number of genes associated with endosperm related traits, for which the functions are unknown.

## Experimental materials and methods

### Plant material

Maize (*Zea mays* L.) inbred line Mo17 was planted and grown under the recommended agronomic guidelines, and self-pollinated at the Wenjiang Research farm of Sichuan Agricultural University. Developing ears at different days after pollination (DAP) were obtained and used in this study.

### Experimental designs

In this study, two designs of response surface method (RSM); Box–Behnken and Central Composite designs (BBD & CCD), were used to model important factors affecting isolation and transfection of MEPs, with a goal to optimizing protoplast yield and transfection efficiency. A central assumption is that the independent variables or factors are continuous and adjustable by experiments with negligible errors. The RSM involves designing of experiments (DOE) to provide suitable and reliable measurements of the response which help the experimental data to fit the response model with use of minimum number of tests. Protoplast system has been established for various plant tissues and species [13, 22, 31]. Among various tissues of the same plant and same tissue-type among plant species, little variations exist for the different isolating factors such as hydrolytic enzymes, mannitol, and duration of hydrolysis. Therefore, the levels of the different isolating factors tested in this study, covered the level-range of each isolating factor reported for maize leaf and nucellus protoplasm [13, 22, 39]. In order to obtain the best settings for the combination of the isolation parameters, we adopted effective statistical and predictive modeling approach, that will find optimal levels of the parameters with high precision. In this study, we used BBD to establish appropriate settings of isolation conditions for quality protoplast yield. This design is commonly used for analysis of factors in three levels, coded as −1, 0, and +1, and requires much fewer tests than the full factorial. The protoplast isolation experiment involved four factors; Cellulase Concentration, Macerozyme Concentration, Mannitol Concentration and Hydrolysis time, coded as X_1_, X_2_, X_3_, and X_4_, respectively. Orthogonal combinations of the experimental units involving the four factors with 5 tests at the center points gave a total of 29 tests, as generated with Design-Expert.8.0.6 software. The coded and actual levels of the variables with the total number of the experimental tests are given in Table 1. The CCD was selected for the optimization of protoplast transfection parameters including protoplast concentration, amount of total plasmid DNA and maize endosperm age; coded as X_1_, X_2_, and X_3_, respectively. The CCD requires 5 levels of each factor: −α, −1, 0, 1, and +α. The protoplast transfection experiment comprised 23 experimental units, formed from orthogonal combinations of the 3-factors with 9 central points, and was designed with Design-Expert.8.0.6 software (Table 2). The required number of experimental tests for each of the RSM design can be determined by Eq. (3)

3 $$2^{\text{k}} + {\text{ 2k }} + {\text{ r }} = {\text{ Number of required experimental runs}}$$

where K = Number of experimental factors or variables and r = Number of tests at the center points.

### Protoplast isolation

The MEPs were isolated based on the methods reported by Chen et al. [13] with minor modifications. Developing maize ears at 8 DAP were harvested and used for the protoplast isolation experiment. Endosperms from different ears were excised, bulked together and digested. The digested sample was divided into three, and each was used for independent experiment. Concisely, tweezers were used to remove the seed coat of the kernels (3–4 mm in diameter), and then developing endosperms were obtained and immediately placed on MS agar medium. The endosperm was gently cut on clean petri-dish using a sharp surgical blade with the aid of forceps, and quickly transferred into 100 ml conical flask containing 10 ml of freshly prepared enzyme solution of each experimental group. About 30–40 endosperms are digested in 10 ml of enzyme solution. The enzyme solutions contained the concentrations indicated in Table 1 for (w/v) cellulase R-10 (Yakult Pharmaceutical), (w/v) macerozyme R-10 (Yakult Pharmaceutical), and mannitol with 20 mM MES (pH 5.7). The enzyme solutions were warmed up to 55 °C for 10 min, and allowed to cool to room temperature before 1 M CaCl_2_ and 0.1% BSA were added and then filtered. The endosperms were completely submerged in the enzyme mixture and allowed to digest in the dark at 26 °C for 4–8 h without agitation. Then, an equal volume of W5 (5 M NaCl, 1 M CaCl_2_, 2 M KCl and 0.2 M MES) was added to the enzyme mixture to stop the hydrolysis, and was vigorously shaken for 5 s to release protoplasts. The protoplast-enzyme suspension was filtered through a 100 µm nylon mesh to remove tissue debris (Note: the mesh is normally kept in 95% ethanol and rinsed with W5 solution before use), and the filtrate was transferred to a 10-ml eppendorf tube. The flow-through was horizontally centrifuged at 110×*g* for 3 min to pellet the protoplasts. The supernatant was discarded, and the protoplasts were re-suspended in 3 ml W5. The suspension was kept on ice for 30 min, centrifuged as above and discarded the supernatant. The protoplast pellet was gently re-suspended in 1 ml MMG (2 M MgCl_2_, 0.8 M Mannitol, 0.2 M MES) for use. All pipette tips used in the protoplast isolation were cut with scissors and autoclaved. The protoplast cells were quantified by microscopy using a hemocytometer. The viability of protoplasts was determined by the FDA staining method [40].

### Plasmid construction, PEG-calcium mediated protoplast transfection and microscopy

The full sequence of GFP was amplified and cloned into pBI221 vector driven by *ubiquitin* promoter to produce pBI221-*GFP*, and was used for protein immunoblotting. We constructed 2300-*ZmBt1*–*GFP* plasmid by cloning the full fragment of ZmBT1 into 2300-*GFP*, to investigate the subcellular localization of ZmBT1 protein. The empty 2300-*GFP* vector was used as control. For BiFC, the coding sequences of endosperm-specific transcription factors O2 and PBF1 were cloned into two-molecule fluorescent complementary expression vectors, E3108 and E2884, respectively, to obtain E3108-*O2* and E2884-*PBF1*. The empty plasmids, E2884 and E3108 were paired with E3108-*O2* and E2884-*PBF1*, respectively, and used as controls. 10 µg of each of the constructed plasmids was separately transfected into the MEP as described below.

A modified PEG-Ca^2+^ mediated protoplast transfection protocol by Yoo et al. [31] with minor modifications was used. The MEP yield was adjusted with MMG solution to the final required concentrations of 0.5–2.5 × 10^6^ cells/ml. Foremost, 5–15 µg plasmid DNA was mixed with 100 µl protoplasts in a 2 mL centrifuge tube. Then, an equal volume (110–120 µl) of freshly prepared PEG-calcium (30% PEG4000, 0.8 M mannitol, 1 M CaCl_2_) transfection solution was added and the solution was gently mixed. The DNA-PEG-calcium-protoplast solution was incubated at 26 °C for 20 min, and 440 µl W5 solution was added, gently mixed and centrifuged at 110×*g* for 2 min. The supernatant was carefully removed, and the pelleted protoplasts were re-suspended in 1 ml W5 solution. The suspension was centrifuged at 110×*g* for 2 min, supernatant discarded and gently re-suspended the pellets in 1 ml W5 solution. The integrity of the protoplasts was examined under microscope after fluorescein diacetate (FDA) staining [40]. The protoplasts were then incubated in the dark conditions at 26 °C for 12–16 h for further experimental analysis.

The transformed protoplasts were incubated with 0.1 μg ml^−1^ 4′,6-diamidino-2-phenylindole (DAPI) for 5 min. 5 µl of protoplast cells containing the GFP fusion proteins was used for fluorescence microscopy. Laser Scanning Confocal Microscope (LSCM) was used for visualization under the Nikon A1Si Laser Scanning Confocal Microscope, at the excitation and emission wavelengths of 488/507, 358/461 and 480/530 nm for GFP, DAPI and FDA, respectively. At least three independent fluorescence experiments were performed. One experiment represent average of five microscopic fields. Transfection efficiency was calculated as the number of fluorescent protoplasts in view divided by total protoplast number in view from one experiment. The percentage of the average of three independent experiments was determined to give the transfection efficiencies.

### Protein extraction and immunoblotting

The transfected endosperm protoplasts (EPs) were harvested by centrifugation at 4 °C, 1000×*g* for 3 min and the supernatant was discarded. Proteins were extracted by boiling in SDS-PAGE buffer [50 mM Tris–HCl (pH 7.5), 150 mM NaCl, 5 mM EDTA, 0.2% NP-40, 0.1% Triton X-100, and Complete protease inhibitor cocktail, Roche] for 10 min. The extracts were centrifuged at 12,000×*g* for 5 min. The supernatants were collected for Western blot analysis. The protein was separated by 10% SDS-PAGE gel electrophoresis and transferred to a PVDF membrane. After incubation with anti-GFP and anti-mouse IgG antibody [Abmart (Shanghai) Co., Ltd], the product was visualized using a chemiluminescent kit (Beyotime Institute of Biotechnology, Jiangsu, China).

### RNA extraction and qRT-PCR analysis

The coding sequence of *ZmMYB14* was cloned into pBI221 vector as described in our previous study [32] to obtain pBI221-*ZmMYB14*. 10 µg of the plasmid was transfected into EP as described above. The transfected protoplast suspension was centrifuged at 4 degrees C, 13,000×*g* for 1 min and the supernatant was discarded. RNA was extracted according to the RNA Extraction Kit manual (TRizol Companion Kit, Beijing Tian Enze Company). Reverse transcription was carried out using the PrimeScript RT reagent Kit (TaKaRa, Japan). Real-time quantitative reverse-transcriptase PCR assays were performed with a CFX96 Real-Time System (Bio-Rad, California, USA). The PCR mixture (a total volume of 10 µl) contained 0.3 µl forward primer, 0.3 µl reverse primer, 1 µl cDNA, 3.4 µl double-distilled H_2_O, and 5 µl TB Green Premix Ex Taq II. The following program was used for the amplification: 95 °C for 30 s, 95 °C for 5 s, 59 °C + Plate Read 30 s, 40 cycles of 95 °C for 5 s, 95 °C for 10 s and melt curve, 65 °C to 95 °C, increase 0.5 °C for 0.05 s + Plate Read. The following primers; 5′-GGTGTTCCAGTGGATCATG-3′ (*ZmBT1QF*), 5′-CCGTGTCATAGGTGAAATG-3′ (*ZmBT1QR*), 5′-CGCACGGATAACGAGGTCA-3′ (*ZmMYB14QF*), and 5′-TGAGTTGAAGTGGGCAGGATTG-3′ (*ZmMYB14QR*) were used for the qRT-PCR. The PCR was performed in triplicates, and relative transcription levels were calculated using the 2^−ΔΔCt^ method. The maize *TXN* gene was used as internal control because its expression level remained relatively constant across endosperm development [41].

### Statistical analysis

The protoplast yields and transfection efficiencies data were analyzed in RSM by Design-Expert.8.0.6 software. Protoplast yield response was analyzed in a quadratic regression model with cellulase, macerozyme, mannitol and hydrolysis time as independent variables. The transfection efficiencies data were transformed according to Eq. (4), before regressing it on protoplast conc., total plasmid DNA and endosperm age. Optimization of protoplast yield and transfection efficiency were performed to determine optimal settings for the independent variables in each case.

4 $${\text{y}^{\prime} } = \, \left( {{\text{y }} + {\text{ k}}} \right)^{ 2}$$

where y′= transformed transfection efficiency and k = 0 (constant)

## Data Availability

All the data generated or analyzed during this study are included within this article.

## Abbreviations

BBD: Box–Behnken design
BiFC: Bimolecular fluorescent complementation
CCD: Central composite design
EP: Endosperm protoplast
EPS: Endosperm protoplast system
ESD: Endosperm development
EtR: Endosperm-trait related
MEP: Maize endosperm protoplast
RSM: Response surface method

## References

[1] Berger F (1999). Endosperm development. Curr Opin Plant Biol.

[2] Brown RC, Lemmon BE (2007). The developmental biology of cereal endosperm. Plant Cell Monogr..

[3] Olsen O (2004). Nuclear Endosperm Development in Cereals and Arabidopsis thaliana. Plant Cell..

[4] Sabelli PA, Larkins BA (2009). The development of endosperm in Grasses. Plant Physiol.

[5] Olsen O, Becraft PW. Endosperm Development. Seed Genomics. 2013;43–62.

[6] Sabelli PA, Liu Y, Dante RA, Lizarraga LE, Nguyen HN, Brown SW, et al. Control of cell proliferation, endoreduplication, cell size, and cell death by the retinoblastoma-related pathway in maize endosperm. Proc Natl Acad Sci. 2013; 110(19):E1827–36. https://www.pnas.org/content/110/19/E1827. 10.1073/pnas.1304903110 PMC3651506 23610440

[7] Huang Y, Wang H, Huang X, Wang Q, Wang J, An D (2019). Maize VKS1 regulates mitosis and cytokinesis during early endosperm development. Plant Cell..

[8] Hannah LC, James M (2008). The complexities of starch biosynthesis in cereal endosperms. Curr Opin Biotechnol.

[9] Hu Y-F, Li Y-P, Zhang J, Liu H, Tian M, Huang Y (2012). Binding of ABI4 to a CACCG motif mediates the ABA-induced expression of the ZmSSI gene in maize (*Zea mays* L.) endosperm. J Exp Bot.

[10] Li Y, Yu G, Lv Y, Long T, Li P, Hu Y (2018). Combinatorial interaction of two adjacent cis-active promoter regions mediates the synergistic induction of Bt2 gene by sucrose and ABA in maize endosperm. Plant Sci.

[11] Liu F, Ahmed Z, Lee EA, Donner E, Liu Q, Ahmed R (2011). Allelic variants of the amylose extender mutation of maize demonstrate phenotypic variation in starch structure resulting from modified protein–protein interactions. J Exp Bot.

[12] Mazarei M, Al-Ahmad H, Rudis MR, Neal Stewart C (2008). Protoplast isolation and transient gene expression in switchgrass, *Panicum virgatum* L.. Biotechnol J.

[13] Chen J, Yi Q, Song Q, Gu Y, Zhang J, Hu Y (2015). A highly efficient maize nucellus protoplast system for transient gene expression and studying programmed cell death-related processes. Plant Cell Rep.

[14] Miao Y, Jiang L. Transient expression of fluorescent fusion proteins in protoplasts of suspension cultured cells. Nat Protoc. 2007;2(10):2348–53. 10.1038/nprot.2007.360. 10.1038/nprot.2007.360 17947977

[15] Bart R, Chern M, Park C, Bartley L, Ronald PC (2006). A novel system for gene silencing using siRNAs in rice leaf and stem-derived protoplasts. Plant Methods..

[16] Zhang Y, Su J, Duan S, Ao Y, Dai J, Liu J, et al. A highly efficient rice green tissue protoplast system for transient gene expression and studying light/chloroplast-related processes. Plant Methods. 2011;7(1):30. http://www.plantmethods.com/content/7/1/30. 10.1186/1746-4811-7-30 PMC3203094 21961694

[17] Priyadarshani SVGN, Hu B, Li W, Ali H, Jia H, Zhao L (2018). Simple protoplast isolation system for gene expression and protein interaction studies in pineapple (*Ananas comosus* L.). Plant Methods..

[18] Zhang H, Liu Y, Wen F, Yao D, Wang L, Guo J (2014). A novel rice C2H2-type zinc finger protein, ZFP36, is a key player involved in abscisic acid-induced antioxidant defence and oxidative stress tolerance in rice. J Exp Bot.

[19] Lee JH, Jin S, Kim SY, Kim W, Ahn JH (2017). A fast, efficient chromatin immunoprecipitation method for studying protein-DNA binding in *Arabidopsis mesophyll* protoplasts. Plant Methods..

[20] Shen Y, Meng D, McGrouther K, Zhang J, Cheng L (2017). Efficient isolation of Magnolia protoplasts and the application to subcellular localization of MdeHSF1. Plant Methods..

[21] Pitzschke A, Persak H (2012). Poinsettia protoplasts—a simple, robust and efficient system for transient gene expression studies. Plant Methods..

[22] Lung S-C, Schoor S, Sigurdson D, Yanagisawa M, Yeung K, Liu MQ, Yeung E, Stasolla C, Sumner MHB (2015). Protoplast isolation and staining. Plant microtechniques and protocols.

[23] Faraco M, Di Sansebastiano G Pietro, Spelt K, Koes RE, Quattrocchio FM. One Protoplast Is Not the Other!. Plant Physiol. 2011;156(2):474–8. http://www.plantphysiol.org/content/156/2/474.abstract. 10.1104/pp.111.173708 PMC3177251 21454800

[24] Crowder AJ, Landgren CR, Rockwood LL (1979). Cultivar differences in starch content and protoplast yields from root cortical explants of Pisum sativum. Physiol Plant.

[25] Chang MM, Loescher WH (1991). Effects of preconditioning and isolation conditions on potato (*Solanum tuberosum* L. cv. Russet Burbank) protoplast yield for shoot regeneration and electroporation. Plant Sci..

[26] Rose R (1980). Factors that influence the yield, stability in culture and cell wall regeneration of spinach mesophyll protoplasts. Funct Plant Biol.

[27] Bahaji A, Ovecka M, Bárány I, Risueño MC, Muñoz FJ, Baroja-Fernández E (2011). Dual targeting to mitochondria and plastids of AtBT1 and ZmBT1, two members of the mitochondrial carrier family. Plant Cell Physiol.

[28] Shannon JC, Pien F-M, Cao H, Liu K-C. Brittle-1, an Adenylate Translocator, Facilitates Transfer of Extraplastidial Synthesized ADP-Glucose into Amyloplasts of Maize Endosperms. Plant Physiol. 1998;117(4):1235 LP–1252. http://www.plantphysiol.org/content/117/4/1235.abstract. 10.1104/pp.117.4.1235 PMC34888 9701580

[29] Zhang Z, Zheng X, Yang J, Messing J, Wu Y (2016). Maize endosperm-specific transcription factors O2 and PBF network the regulation of protein and starch synthesis. Proc Natl Acad Sci.

[30] Sheen J (2001). Update on signal transduction signal transduction in maize and arabidopsis mesophyll protoplasts. Plant Physiol.

[31] Yoo SD, Cho YH, Sheen J (2007). Arabidopsis mesophyll protoplasts: a versatile cell system for transient gene expression analysis. Nat Protoc.

[32] Xiao Q, Wang Y, Du J, Li H, Wei B, Wang Y (2017). ZmMYB14 is an important transcription factor involved in the regulation of the activity of the ZmBT1 promoter in starch biosynthesis in maize. FEBS J.

[33] Sun B, Zhang F, Xiao N, Jiang M, Yuan Q, Xue S (2018). An efficient mesophyll protoplast isolation, purification and PEG-mediated transient gene expression for subcellular localization in Chinese kale. Sci Hortic (Amsterdam)..

[34] Mathur J, Koncz C, Salinas J (1998). PEG-mediated protoplast transformation with naked DNA. Arabidopsis protocols in methods in molecular biology.

[35] Kowles RV, Phillips RL (1988). Endosperm Development in Maize. Int Rev Cytol.

[36] Zhan J, Dannenhoffer JM, Yadegari R. Endosperm Development and Cell Specialization. In: Larkins BA, editor. Maize Kernel Development. CAB International; 2017. p. 28–43. https://lccn.loc.gov/2017023565.

[37] Lung SC, Yanagisawa M, Chuong SDX (2011). Protoplast isolation and transient gene expression in the single-cell C4 species, *Bienertia sinuspersici*. Plant Cell Rep.

[38] Reyes FC, Sun B, Guo H, Gruis D (Fred), Otegui MS. Agrobacterium tumefaciens Mediated Transformation of Maize Endosperm as a Tool to Study Endosperm Cell Biology. Plant Physiol. 2010;153(2):624 LP–631. http://www.plantphysiol.org/content/153/2/624.abstract. 10.1104/pp.110.154930 PMC2879798 20357137

[39] Gao L, Shen G, Zhang L, Qi J, Zhang C, Ma C (2019). An efficient system composed of maize protoplast transfection and HPLC–MS for studying the biosynthesis and regulation of maize benzoxazinoids. Plant Methods..

[40] Larkin PJ (1976). Purification and viability determinations of plant protoplasts. Planta.

[41] Li G, Wang D, Yang R, Logan K, Chen H, Zhang S, et al. Temporal patterns of gene expression in developing maize endosperm identified through transcriptome sequencing. Proc Natl Acad Sci. 2014;111(21):7582 LP–7587. http://www.pnas.org/content/111/21/7582.abstract. 10.1073/pnas.1406383111 PMC4040564 24821765

